# Time-resolved cathodoluminescence of DNA triggered by picosecond electron bunches

**DOI:** 10.1038/s41598-020-61711-x

**Published:** 2020-03-19

**Authors:** Jean Philippe Renault, Bruno Lucas, Thomas Gustavsson, Alain Huetz, Thomas Oksenhendler, Elena-Magdalena Staicu-Casagrande, Marie Géléoc

**Affiliations:** 1 Université Paris-Saclay, CEA, CNRS, NIMBE, 91191 Gif-sur-Yvette, France; 2 Laboratoire de Physique des Gaz et des Plasmas, CNRS, Université Paris-Saclay, Université Paris-Sud, 91405 Orsay, France; 3 Université Paris-Saclay, CEA, CNRS, LIDYL, Gif-sur-Yvette, 91191 France; 4 Institut des Sciences Moléculaires d’Orsay, CNRS, Université Paris-Saclay, Bât. 520, Université Paris-Sud, 91405 Orsay, cedex France; 5 ITEOX, 14 Avenue Jean Jaurès, 91940 Gometz-le-Châtel, France

**Keywords:** Chemical physics, Biological physics

## Abstract

Despite the tremendous importance of so-called ionizing radiations (X-rays, accelerated electrons and ions) in cancer treatment, most studies on their effects have focused on the ionization process itself, and neglect the excitation events the radiations can induce. Here, we show that the excited states of DNA exposed to accelerated electrons can be studied in the picosecond time domain using a recently developed cathodoluminescence system with high temporal resolution. Our study uses a table-top ultrafast, UV laser-triggered electron gun delivering picosecond electron bunches of keV energy. This scheme makes it possible to directly compare time-resolved cathodoluminescence with photoluminescence measurements. This comparison revealed qualitative differences, as well as quantitative similarities between excited states of DNA upon exposure to electrons or photons.

## Introduction

Most studies on the effect of so-called ionizing radiations (X-rays, electrons, ions) have focused on the ionization process itself. However, ionizing radiation can also efficiently induce excitations in various molecules, whose effects are rarely studied in radiation chemistry and biochemistry. In particular, there is very little literature on the excited state dynamics of DNA following the initial energy deposition by charged particles. For example, highly energetic excited states can be produced by energetic electrons (as suspected from electron energy-loss spectroscopy on DNA^1,2^, showing a “plasmon band” at 25 eV) with expected, associated short lifetimes^3^, and putative damaging effects when they cause molecular reorganization. Furthermore, such states may migrate, potentially changing the dose distribution locally.

One way to identify and study such states is to analyse their luminescence. The luminescence of DNA under ionizing radiations (also called scintillation) has been reported in the literature – albeit scarcely — especially at low temperatures. For instance, in frozen DNA, a microsecond 4.3 MeV electron excitation induced a “short-lived“ emission extending up to 530 nm^4^. More recently, “in-pulse” nanosecond (ns) luminescence spectra of irradiated (260 keV electrons) wet and dry DNA^5–7^ showed the same broad emission that is sensitive to the presence of energy or electron traps. A negligible amount of light is emitted after the ns pulse, leading to the suggestion that excited states form by geminate recombination of electrons and holes and/or by direct excitation, with sub-ns relaxation times. Further progress on this topic has been impaired by the lack of appropriate time-resolved sources of ionizing radiation. The so-called pulsed radiolysis technique relies on a high-energy particle accelerator, which has two drawbacks^4^: (i) it has a low repetition rate (a few hundred Hz at the most) that is not compatible with ultrafast time-resolved techniques, such as time-correlated single photon counting, and limits the time resolution to the ns range^8^; (ii) it provides high-energy beams that produce Cerenkov light, which blinds the detector^9,10^. Here, we describe a table-top, high-repetition-rate, pulsed electron beam system that is compatible with most kHz to MHz femtosecond (fs) lasers, and we use this device to analyse the sub-ns cathodoluminescence (CL), i.e. the luminescence induced by electrons, of genomic DNA and compare it to the photoluminescence (PL) of the same samples. We used this system to answer the following questions: (i) Can ionizing radiations (electrons) efficiently form excited states in dry DNA compared with UV photons?; (ii) Are the same excited states formed by the two methods? (iii) Are the excited states formed directly or by recombination of charge carriers?

## Results and discussion

### The table-top pulsed electron beam system

**Figure 1:**
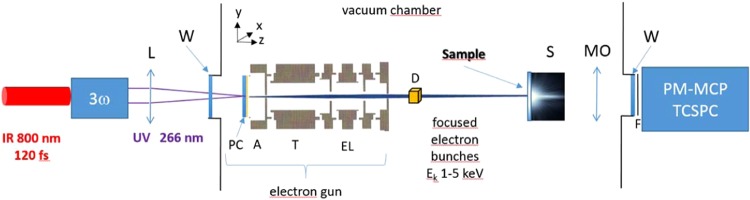
Scheme of the experimental set-up. For the photon part: IR, laser beam at 800 nm; 3w, frequency tripler; UV laser beam at 266 nm; L, optical focusing lens (f = 200 mm); W, uncoated UV fused silica (FS) window; S, sample positioned on a xz motorized table; MO, microscope with UV-VIS reflective objective; F, optical filter; PM-MCP, microchannel plate photomultiplier tube; TCSPC, time-correlated single photon counting. For the electron part: Electron gun: PC, photo cathode; A, drilled anode; T, drift tube; EL, Einzel electronic lens; D, xy deflecting system. An in-house designed Faraday cup coupled to a pico-amperemeter, not shown in the scheme, can be inserted between D and S to monitor the electron beam current, in the pA to nA range. The set-up between the UV FS windows (W) is under vacuum (typically 10^−6^ mbar).

The experimental set-up is based on the use of an ultrafast^11,12^ pulsed electron gun designed in house (Fig. 1). It is based on a photocathode consisting of a gold layer (manufactured at the C2N technology facility, RENATECH) deposited on a sapphire substrate, photo-triggered by a high- repetition-rate fs laser at 800 nm converted to 266 nm. The thickness of the photocathode is around 10 nm, which allows the production of photoelectrons of very low kinetic energy and low energy dispersion^11^. This photocathode also makes it possible to use the very same setup for photoluminescence experiments with excitation at 266 nm, by simply setting the accelerating voltage to 0 and letting through the UV excitation beam (Fig. 1).

An accelerating field directed towards an anode drilled with a small hole and a three-element cylindrical Einzel lens ensure spatial focusing. The optimization of the field lines around the anode was performed using SIMION 8 to minimize the divergence of the beam after passing through the anode and to obtain a spatial resolution better than 50 μm at the chosen focal point (see Supplementary Figure S1).

The pulsed electron gun can be used for average electron current from the fA to the nA range, and for electron energies from 100 eV to 10 keV. However, the maximum electron energy was set to 4.1 keV, a value usually considered safe in terms of radiology protection. Although the energy resolution is not critical in irradiation experiments, the temporal resolution of the electron bunch is. The temporal resolution is first linked to the resolution of the triggering laser pulse. However, in the case of an electron bunch containing many electrons, the bunch broadens during propagation due to Coulomb force and space charge effects. The current is the product of three factors: the number of electrons per bunch, the charge of an electron and the repetition rate of the laser. The use of a 76 MHz laser with low energy per pulse (800 nm, 120 fs, 30 nJ per pulse, converted to 267 nm with an in-house tripler system) limited the number of electrons per bunch and thus minimized the space charge effects, and kept the total current received by the sample high enough to keep acquisition times reasonable.

The initial velocity dispersion and the Coulomb force within the bunches tend to enlarge the electron bunches both in time and position in a rather complex way. In most previous pulsed electron studies, time resolution remained purely nominal, e.g. based on SIMION calculations. Only recently have the first direct measurements become possible^13^. Here, in addition to SIMION 8 simulations, we performed ultrafast streaking measurements (see Supplementary Figures S2–S5), synchronized by a photoconductor in the ELYSE laser facility (ICP, Université Paris Saclay, 790 nm, 1 kHz, 130 fs, 1 mJ converted to 264 nm with an in-house tripler system). The use of a kHz repetition-rate laser instead of a MHz laser was required due to the limited recurrence of the fast streaking system. Moreover, in the ELYSE laser facility, the energy per impulsion can be varied over a large range, and the number of electrons per bunch can be varied accordingly. Apart from their repetition rates, the kHz and MHz laser systems were comparable in terms of wavelength (265 ± 2 nm) and pulse duration (respectively 130 and 120 fs). Beam size and energy per impulsion of the UV photon beam was typically adjusted to 1 mm diameter and below on the photocathode and to 50 pJ and above for the energy per impulsion, to obtain on average one electron or more per bunch.

**Figure 2:**
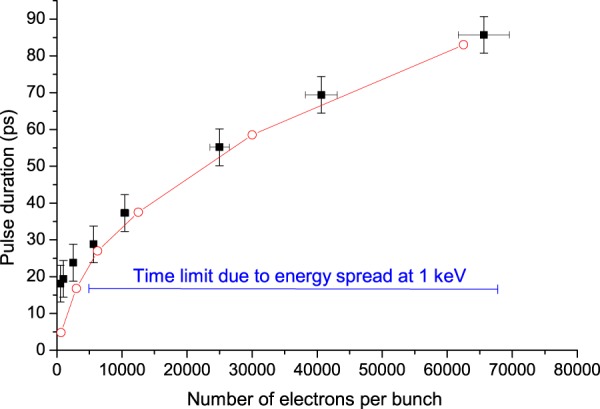
Computed (in red) and measured (in black) electron bunch temporal width as a function of the number of electrons per bunch, for 1 keV electrons and for a 600 μm laser beam diameter on the photocathode.

The time resolution achieved by the streaking system for 1 keV electrons was below 5 picosecond (ps) on the sample at the focal point. At 1 keV, with 5.10^2^ electrons per pulse, the measured electron bunch duration (18 ± 5 ps) was mainly determined by the initial energy spread (see Supplementary Information part A.2. and Fig. 2). At the energy of 4.1 keV and 10^2^ electrons per pulse used in the luminescence experiments described below, simulations showed that the bunch duration was shorter, typically in the 5 ps range, owing to more limited Coulomb force and energy spread effects in these conditions.

The beam size on the sample was voluntarily larger than that of electron microscopy-based (SEM and TEM) ultrafast cathodoluminescence systems^14^ so as to minimize the dose received by the sample. A gold layer on the top of the sample (40 nm) was also used to control the dose, and to ensure optical insulation and the evacuation of the electrical charges. The electron beam size was evaluated at 500 µm on the sample using the knife-edge technique (see Supplementary part A.1.). The penetration depth of the beam was evaluated at 150 nm using CASINO software^15^ (see Supplementary Figure S6). These geometrical considerations give a dose rate in the kGy.s^−1^ range for the sample, compared to tens of MGy.s^−1^ in TEM systems (see Supplementary Information part A.3. and refs. ^16,17^).

### Electron-induced compared to UV-induced luminescence

As stated above, a gold film was deposited on top of the samples, to limit the dose and prevent charging. To check that this gold film did not induce luminescence either by plasmon excitation or by transition radiation, we deposited it on a bare sapphire substrate.

The luminescence detection system was then tested using a commercial fast scintillator, BC-422Q^18,19^, in which the excited states created by the ionizing radiation are partially quenched by the addition of benzophenone (5%). It has a nominal 0.7 ns time constant^20^.

**Figure 3:**
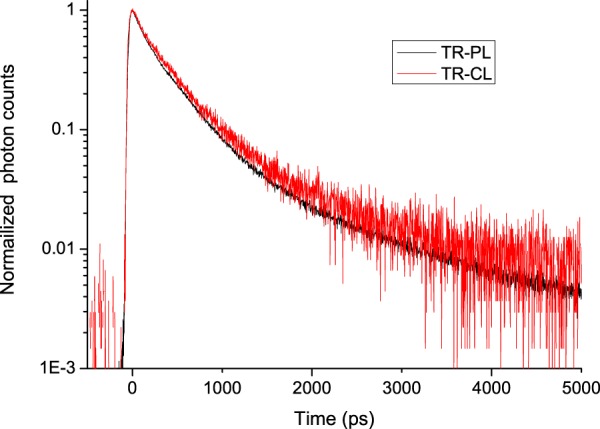
Comparison of time-resolved cathodoluminescence (TR-CL) and photoluminescence (TR-PL) of the BC-422Q scintillator.

We compared direct laser excitation at 266 nm (time-resolved photoluminescence, TR-PL) and electron excitation (time-resolved cathodoluminescence, TR-CL) of BC-422Q (Fig. 3). Of course, in the latter case, no light was observed in the absence of acceleration voltage. In the case of UV excitation, no reliable luminescence data was obtained around the excitation wavelength. Therefore, both decay curves are presented with emissions blocked below 300 nm. In our measurements, comparable luminescence lifetimes and spectra of BC-422Q were obtained with UV or electron excitation. These lifetimes and spectra (Figures S7–S9) are well-described by a tri-exponential model function relaxing in 100 ps, in 400 ps and about 1 ns respectively as shown by the fitted curve in Supplementary Figure S8. The values measured here seem to be on average lower than those given by the manufacturer, but are relatively comparable to those measured by coincidence at 200 and 380 ps^21^. For comparison purposes, the UV excitation data were not deconvoluted for the instantaneous response function, because this function cannot be measured for the electron beam (this would require a scintillator with a calibrated lifetime in the ps range).

A rough insight into the TR-CL emission spectrum was given using low-pass optical filters. Owing to the limited collection efficiency of the detection system and to the low luminescence of the samples we used, the present setup did not allow for the measurement of full-time and wavelength-resolved luminescence spectra. Compared with TR-CL, TR-PL showed an additional emission centred between 300 and 500 nm, with a ns lifetime (Supplementary Figures S7 and S9) and a significant contribution to the total emission.

In the classic view of scintillation processes, ionizing radiation can excite molecules (M) into higher excited singlet (^1^M**) states. The involved particle can also ionize molecules to molecular ions (M^+^) and electrons (e^-^). Their subsequent recombination can also yield ^1^M** or ^3^M**. M** can undergo internal conversion to lower excited states (M*) that will follow traditional photophysical processes (light emission, excimer formation, energy transfer, charge transfer, etc.). In an organic scintillator, this internal conversion may be inefficient, leading to a slow increase in emission, not observed here^22^. In BC-422Q scintillation, an additional step is involved because the light emission comes from energy transfer of M* states to dissolved fluorescent dyes, probably PPO^23^. The expected lifetime measured is then the one of excited dye in the presence of benzophenone^22^, which explains the similarities between the lifetimes observed in TR-PL and TR-CL. The sole specificity in our experiments is the slow emission in TR-PL between 300 and 500 nm. This specificity may arise from the formation of PPO excimers^24^, probably in connection with the different ionization densities of UV photons compared with the electron tracks^25^.

To test the ability of our system to resolve the weak cathodoluminescence from nucleic acids, we turned to spin-coated genomic DNA films (see Methods). These films were prepared from DNA–cationic surfactant complexes so as to be transparent and withstand high vacuum conditions^26^.

**Figure 4:**
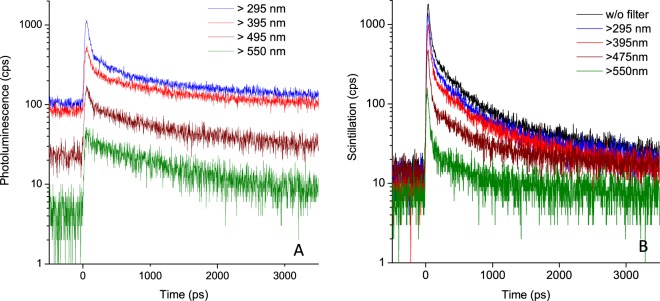
(**A**) Time-resolved photoluminescence of DNA film (TR-PL) and (**B**) time-resolved cathodoluminescence (TR-CL) of the same type of DNA film. The photoluminescence data could not be obtained without filter due to the strong 266 nm background.

Their TR-CL and TR-PL properties are compared in Fig. 4. The CL and PL decays clearly differ significantly, contrary to what was observed for the scintillator. Moreover, in both cases, the luminescence decays were characterized by the presence of an ultrafast component. The TR-PL of DNA can be described with a bi-exponential model function (Fig. 4A and Table 1), having a minor component centred in the UV range and characterized by a lifetime τ_1_ of about 60 ps, and a major component whose lifetime τ_2_ increases from 600 ps in the 300–400 nm wavelength range to 1.1 ns above 550 nm. The fast component τ_1_ represents only 15% of the emitted photons. No TR-PL data are available in the literature on similar DNA films, but it is instructive to compare our results with solution phase data. Natural DNA in solution has a low fluorescence yields of about 10^−4^ ^27,28^. TR-PL of natural DNA (purified genomic calf thymus DNA, reported in 2010^27^) spans five decades of time, ranging from fs to ns, and 98% of the photons are emitted beyond 10 ps. This fluorescence of DNA contrasts greatly with the TR-PL of monomeric chromophores and model helices. Although the TR-PL of the former occurs on a sub-ps time-scale^29^, that of double-stranded model helices ranges typically from about 1 to 10 ps^30^. The exceptionally long-lived and emission-wavelength-dependent TR-PL of natural DNA has been attributed to “delayed” fluorescence, due to repopulation from long-lived “dark” states such as excimers, exciplexes and charge-transfer states^27^. The existence of such long-lived states has been shown independently using transient absorption^31^. Our TR-PL observations thus correspond qualitatively to what has been observed in solution.

**Table 1:** Temporal and intensity analysis of the time-resolved emission in Fig. 4.

	TR-PL		TR-CL	
	Fast component lifetime τ_1_ (% of emitted photons)	Slow component lifetime τ_2_ (% of emitted photons)	Fast component lifetime τ_1_ (% of emitted photons)	Slow component lifetime τ_2_ (% of emitted photons)
Whole wavelength range (185 to 800 nm)	Not studied	Not studied	30 ps (22%)	0.46 ns (78%)
>295 nm	45 ps (13%)	0.63 ns (87%)	35 ps (24%)	0.43 ns (76%)
>395 nm	60 ps (10%)	0.96 ns (90%)	35 ps (20%)	0.49 ns (80%)
>475 nm	44 ps (7%)	0.93 ns (93%)	25 ps (26%)	0.46 ns (74%)
>550 nm	n.d. (<5%)	1.1 ns (>96%)	30 ps (28%)	0.50 ns (72%)

The distinction between the fast and the slow component comes from the bi-exponential fit of the decay in Fig. 4. The relative errors of the lifetime vary from 1% for the whole wavelength range to 10% for the noisiest curves (>550 nm). For the % of emitted photons, the absolute error was evaluated at 3%.

The TR-CL of DNA films, (Fig. 4B and Table 1) observed here contains an ultrafast component, which is faster than the resolution of the detection system (τ_1_ < 30 ps) and a longer major component (τ_2_ of 470 ± 40 ps). Both time constants are wavelength-independent, within experimental errors. The ultrafast component accounts for about one-fourth of the emitted photons. Our results confirm and supplement the various cathodoluminescence studies of DNA^5–7^, showing that there is indeed an ultrafast emission upon exposure to ionizing radiations.

**Figure 5:**
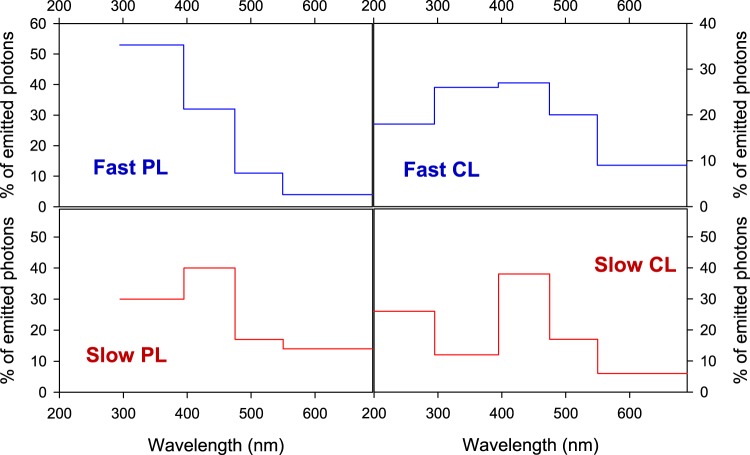
Distribution of the photons emitted by DNA as a function of the wavelength range for the two excitation techniques (PL, photoluminescence; CL, cathodoluminescence). In PL data, the photons below 300 nm were not accessible because the excitation occurs in this wavelength range. The absolute error of the % of emitted photons was evaluated at 3%.

A comparison of TR-CL and TR-PL properties of the same sample (Figs. 4 and 5, Table 1) shows that: (i) the emission spectra are different for the fast and slow components for both CL and PL; (ii) the emission spectrum of the fast component in CL is shifted towards the red compared to that of direct UV excitation, (iii) the fast component accounts for a greater proportion of emitted photons in the CL case than in the PL one.

There are three possible explanations for these different behaviours:

The first is that, despite our precautions, electron excitation causes radiation damage, changing both the emission and relaxation properties of the DNA sample (see dose considerations above). The four main forms of radiation damage on dry DNA are 8-oxoguanine (8OG), 2,6-diamino-4-hydroxy-5-formamidopyrimidine (FapyG), dihydrothymine (DHTy) and strand breaks^32^. None of the modified bases (8OG, FapyG or DHTy) has known emission properties compatible with the emissions observed. 8OG has weak fluorescence located at 350 nm about twice as strong as that of dGMP^33^. The excited state lifetime of 8OG is only slightly longer than that of dGMP, both on the sub-ps time scale^34^. FapyG and DHThy have no reported fluorescence or luminescence. Strand breaks, however, will disrupt base organization and electronic couplings locally and may thus facilitate the formation of new electronic states with different photophysical properties. A red-shifted emission band has been observed upon intermediate fluence excitation of DNA at 266 nm^27^ and interpreted as coming from an excimer-like band. Thus, such damage should also appear in TR-PL, implying that the CL and PL luminescence decays/spectra should be similar, contrary to what we observe.

The second explanation is the excitation of different types of sequences by the two methods: poly(dAdT)·poly(dAdT) has a significantly red-shifted emission compared with poly(dGdC) poly(dGdC), probably due to excimer states^28^. However, the PL of natural DNA is very similar to that of a stoichiometric mixture of monomeric bases^27^.

The third explanation is that electron excitation leads to the population of low-lying, fast-relaxing states that are inaccessible by direct UV irradiation, but accessible to electron impact, either through a direct process^35–38^, geminate recombination^39,40^ or due to ionization density. However, such states characterized by higher multiplicities or strong charge separations are usually non-radiative and must therefore rapidly populate “bright” states responsible for the observed luminescence.

These two latter explanations are not mutually exclusive. We can only notice that we do not observe the temporal signature of geminate recombinations or of internal conversions, i.e. a delayed build-up of the emission^22,41^.

We also tried to compare the respective emission yields between TR-CL and TR-PL. Using the scintillator BC422Q as a reference, (see Supplementary Information part C), the scintillation yield of DNA could be evaluated to 12 ± 6 10^−3^ photons per 100 eV absorbed, with the units traditionally used in radiation chemistry. This yield can be compared to the fluorescence quantum yield of nucleic acid, which is on the order of 3*10^−4^ for 4.5 eV photons at room temperature^28^. This value converts into 6.6 ± 0.6*10^−3^ photons per 100 eV absorbed by DNA. For the same amount of energy injected into the DNA, light emission seems as efficient for accelerated electrons as for UV photons. This similarity does not necessarily mean that excited states are created by electrons as efficiently as by UV photons, but the proximity in the average lifetimes between TR-CL and TR-PL does strongly suggest comparable excitation rates.

An associated question is whether these excited states are of importance in exposure to ionizing radiations. An excess of damage has been identified with respect to trappable radical and excited states have been proposed as a possible explanation of this excess^40,42^. Our calculations do not provide the exact yield in excited states, but we know that the quantum yield of the major damages (chromophore loss) arising from these excited states is 20 times the light emission yield with 254 nm photons^43^. In the case of DNA excitation by electrons, this translates to 0.24 ± 0.12 potential damage sites per 100 eV. This yield can be also compared to the ionization damage yields in dry DNA, respectively on the order of one damage site per 100 eV for 8OG, and 0.2–0.6 respectively for FapyG and DHTy^44^. The effect of electron-induced excitation is therefore not completely negligible. Its role may be even more important in the formation of complex damage, the more toxic type of damage, because excitation may tend to relocalize on already damaged sites, which can act as energy traps.

## Conclusion

The development of a well-characterized table-top ultrafast (photo)electron gun gave new insight into the behaviour of DNA under ionizing radiation. Our results show that i) keV electrons are as efficient as UV photons to form excited states in dry DNA; ii) the excited states formed by keV electrons are lower in energy than those formed by UV photons and show faster decay; iii) there is no indication that the excited states formed by keV electrons arise from geminate recombination.

The chemical effect of these excited states has to be taken into account in the primary yield of DNA damage, but the various energy transfers they can withstand can also change the spatial distribution of the radiolytic events.

Whereas similar sources currently developed in the world are mainly applied to ultrafast electron diffraction (UED) and microscopy, we demonstrate that this type of source is relevant for experiments where photon pulses are replaced by short electron bunches, using an instrument much simpler and much less expensive compared with facilities providing pulsed high-energy particles. For future research, the low-light emission yields may make it possible to perform time-resolved Raman spectroscopy experiments, with minimal modifications of the setup, to identify reaction intermediates based on their vibrational signature. In the long run, a second gun of a similar design may be added in grazing incidence to probe the evolution of the diffraction signature of the sample after ionization in a time-resolved fashion. The combination of these techniques may provide a more complete picture of the process occurring after the interaction of the electron bunches with DNA. The sample environment can also be improved, to allow working with circulating liquid samples. These improvements are now possible with liquid cells by working with ultrathin windows under vacuum. Our results also suggest studying the scintillation of fixed/frozen biological samples, e.g. in SEM/TEM equipped with cathodoluminescent imaging, to identify the differences in the scintillation response of DNA as a function of its location inside nuclei and degree of condensation.

## Methods

### Laser source and PL or CL detection system

The laser source was a commercial mode-locked Ti:Sapphire (MIRA 900, Coherent), pumped by a cw solid state laser (VERDI V10, Coherent), delivering ~120-fs pulses at 800 nm at 76 MHz repetition rate. The maximum pulse energy at 800 nm is 27,6 nJ. The IR pulses were frequency-tripled in a tripler designed in house using two BBO type I crystals, with a conversion efficiency of 5 × 10^−2^. Typically, about 100 mW at 267 nm can be attained but a much lower power was used for the actual experiments. (The spectral range is narrowed and centered on 267 nm thanks to the mirrors used in the tripler system). The 267 nm excitation beam was typically attenuated to 15 mW, transported and finally focused by a 200 mm focal length lens on the electron gun photocathode. The direct laser beam was used to trig the electron gun and then blocked (CL: mode 1), or its part transmitted through the photocathode was directed towards the sample (PL: mode 2) positioned inside the chamber. The two modes of luminescence were used distinctively. The luminescence from the sample was collected with a reflective microscope objective (X15, Newport) and passed through the uncoated UV fused silica chamber window and chosen low-pass filters (Schott series WG280, to GG550) before detection. No coating allows avoiding spectral cut in the emitted signal. The optical collection system was inserted into a black tube in order to protect it from diffused UV photons issued from the laser, the transmitted laser beam through the photocathode was stopped in a block beam and the electron beam was deviated from the transmitted laser beam with the deflecting system to obtain a photon background signal as low as possible, down to 1 counts per second (cps). For the time-resolved measurements, a SPC630 card (Becker & Hickl) was used. The triggering was obtained by measuring the IR pulse train with a fast photodiode (ThorLabs PD10) and the luminescence was measured with a cooled MCP-PMT (R3809U-50 and C10373, Hamamatsu). The detector was maintained in the dark for better signal to noise ratio. This combination provides an instrumental response function of ~30 ps (rmsd). The system is controlled by a homemade LabView program.

The photoluminescence system has been described previously^45^.

### Sample preparation

The scintillator (20*20*2 mm^3^) was purchased from Saint Gobain. The DNA film was prepared following^46^ and^26^. The DNA source was salmon testes DNA (Sigma Aldrich ref D1626). It was dissolved at a concentration of 4 g.L^−1^, dialyzed and fragmented by ultrasonic shearing at 200 kJ in an ice bath using a vibracell sonicator. The expected fragment size are about 100–400 base pairs^47,48^. An equal volume of Cetyltrimethylammonium bromide (Sigma Aldrich) at 4 g.L^−1^ was added to induce the precipitation of a DNA- Cetyltrimethylammonium complex. The precipitate was recovered by filtration on quartz QMA filters (Whatman). To prepare the transparent films, the precipitate was dissolved in isobutanol (5% w/v) and spin coated at 800 rpm on a 3 mm thick sapphire window (Thorlabs). The obtained film was transparent in the visible part of the spectrum and had an optical density of 1 at 260 nm. The corresponding film thickness was of 6 µm^26^. The A_260nm_/A_280nm_ ratio was of 1.7 and the A_260nm_/A_230nm_ ratio of 1.9, showing minimal protein contamination in the solid state. The samples were metallized with an Emitech K575X gold sputterer equipped with a quartz microbalance.

## Supplementary information

supporting information.

## Data Availability

The datasets generated during and analysed during the current study are available from the corresponding authors on reasonable request.
