# Supplementary material for: Time-resolved cathodoluminescence of DNA triggered by picosecond electron bunches
Source: Sci Rep. 2020 Mar 19;10:5071. doi: 10.1038/s41598-020-61711-x (PMC7081262; doi:10.1038/s41598-020-61711-x)
Supplement: Supplementary file 1 — supporting information. [file 41598_2020_61711_MOESM1_ESM.docx]

Supporting information for

Time-resolved cathodoluminescence of DNA triggered by picosecond electron bunches

Jean Philippe Renault ^1^, Bruno Lucas ^2^, Thomas Gustavsson ^3^, Alain Huetz ^4^, Thomas Oksenhendler ^5^, Elena-Magdalena Staicu-Casagrande ^4^, Marie Géléoc ^3^

(1) NIMBE, IRAMIS, DRF, CEA, CNRS, Université Paris-Saclay, Gif-sur-Yvette, 91191, France

(2) Laboratoire de Physique des Gaz et des Plasmas, CNRS, Université Paris-Saclay, Université Paris-Sud, Orsay, France.

(3) LIDYL, IRAMIS, DRF, CEA, CNRS, Université Paris-Saclay, Gif-sur-Yvette, 91191, France

(4) Institut des Sciences Moléculaires d’Orsay, CNRS, Université Paris-Saclay, Bât.520, Université Paris-Sud, F-91405 Orsay cedex, France.

(5) ITEOX, 14 Avenue Jean Jaurès, 91940 Gometz-le-Châtel, France

**A. Electron gun and electron beam characterization**


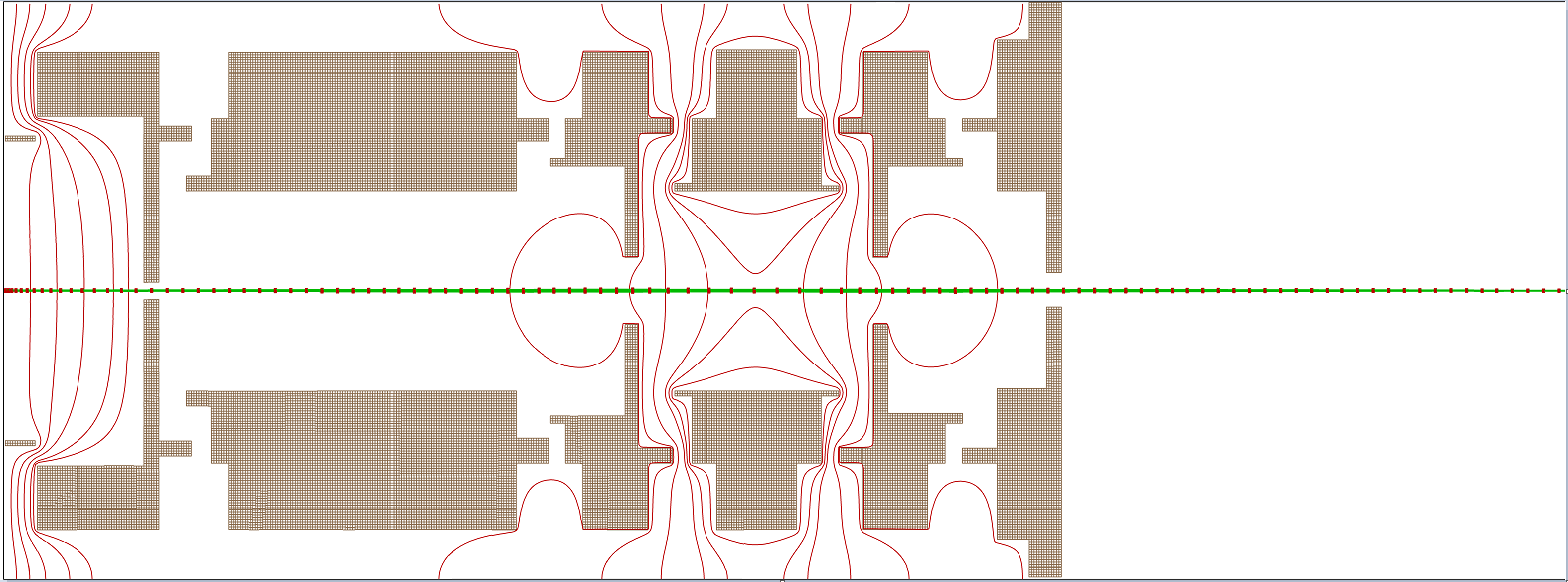


Fig S1: LUBIOL electron gun, sectional view with equipotential lines from SIMION 8 simulation and for an acceleration field leading to 1keV electrons. The electron beam propagation is in green and the red points show the position of the bunches with a 100 ps time step. The distance between the photocathode and the anode is 17 mm, the anode thickness and hole diameter are 3 mm and 2mm respectively and the system length from photocathode at the entrance to the exit hole is 128 mm.

All experiments were conducted with a vacuum chamber at 10^-6^ mbar, including dedicated diagnostics for the characterization and the monitoring of the performances of the source. Emittance, i.e. the number of electron per bunches, was measured with a homemade Faraday cup coupled to a pico-Amperemeter (Keithley). Currents ranging from pA to nA were detected, corresponding to 0.1 electrons per bunch to 100 electrons per bunch with the MHz laser. The overall setup is presented in Fig. S1.

**A.1. Beam size**

The electron beam size on the sample was measured using the knife-edge method to 500 µm, directly from the luminescence data. The light intensity as a function of the edge position was analyzed using a convolution of a Gaussian function (beam profile) by a stair (edge). Source radius is driven by the laser spot size on the photocathode, this is why special care was given to the optical design upstream.

**A.2. Beam temporal structure**

Electron bunches duration measurements were conducted on the ELYSE facility (LCP, Paris-Sud University), where the 1 kHz Ti:Sa laser was coupled to our UV triggered electron gun. The electron beam temporal structure was measured using a specifically developed ultrafast streaking system (iTEOX), triggered with a variable delay controlled by photoconductive switches.

The measurement technique (Fig. S2, S3, S4) is based on the streaking of the electron bunch in a little domain between two plates used to submit the bunch to a time dependent electric field, perpendicular to the bunch propagation. The fast voltage ramp applied to the plates is monitored by lighting the photoconductive switches with the IR laser beam, and thus synchronized with the electron bunch. The first and last arrived electrons of each bunch are submitted to different electric fields. The bunch axis is thus rotated and its projection perpendicular to the propagation axis is the signature of the temporal width of the bunch. The later can then be deduced from a spatial measurement of the electron beam spot imaged on the detector.


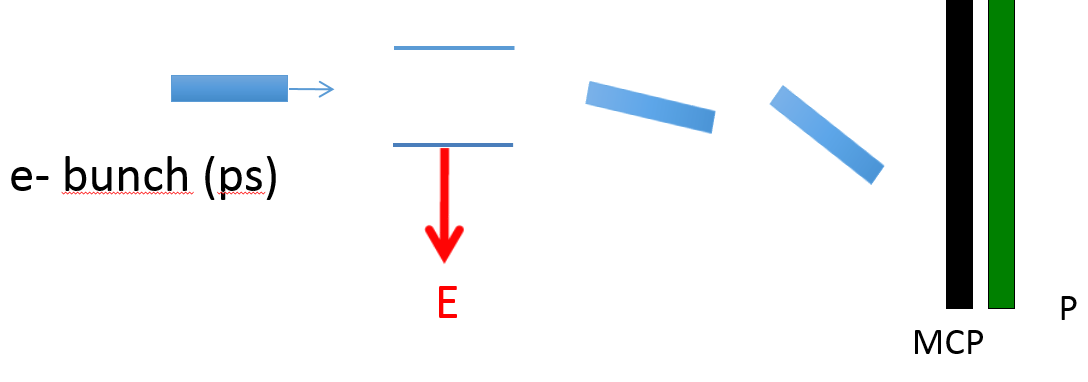


Fig S2 : Measurement principle, E(t) time dependent Electric field, MCP Multi Channel Plate, P Phosphor Screen.


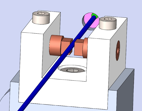


Fig S3 : Detail of the two plates mounted in Fig S2 issued from CATIA modelisation.

The detector consisted in a MCP coupled to a phosphor screen imaged with a lens on a camera (Beamage CCD23, Gentec-EO). For these measurements the focal point of the electron beam was adjusted to be in the middle of the deflecting plates. The streaking system was installed near the tripler system. A beam of 160 μJ was extracted from the main IR beam and sent on a delay line of 300 mm travel range. Then the beam is expanded with a diverging lens to cover the whole surface of one photoconductive switch. The temporal synchronization between the electron bunch and the voltage ramp is roughly tuned from a first estimation of their relative position via TTL signals and then precisely tuned by acting i.) on the cables lengths connecting the plates to the photoswitches and ii.) on the delay line, step by step.


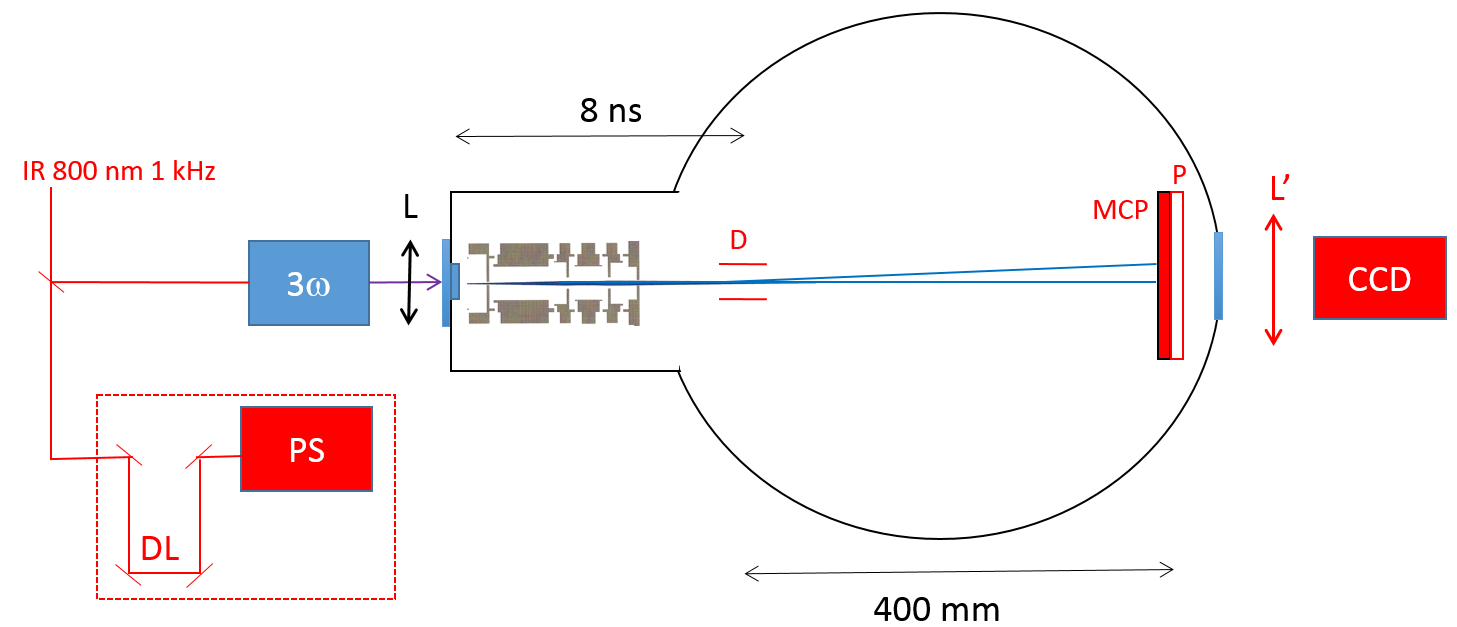


Fig S4: Scheme of the experiment used at ELYSE facility. The elements in red are used for the ultrafast streaking: IR laser beam at 800 nm, DL Delay Line, PS Photoconductive switches, D x deflecting plates, L and L’ optical focusing lens, MCP microchannel plate, P Phosphor screen, CCD camera. Each deflecting plate is connected to one photoconductive switch monitoring the voltage ramp to be applied. Typical time of flight for 1 keV electrons is of 8 ns between the photocathode and the deflecting plates.

The design followed the principles described by Jaanimagi ^1^.

The deflection angle by parallel plates is typically

$$\alpha=LV_{d}/2dV_{a}$$

Where L is the plate length, d their spacing, V_d_ the potential between the plates (up to 400 V) and V_a_ the electron acceleration potential.

Larger plates lead to larger deviation and therefore to a better resolution. However, this formula does not take into account the electron beam size. Indeed, the electrons located at the top and bottom of the beam will not feel the same potential. It induces a temporal shearing of the electron packet, that will ultimately limit the system resolution and that can be evaluated as :

$$\tau_{d}=wLV_{d}/v_{a}dV_{a}=2\alpha w/v_{a}$$

where v_a_ is the average axial speed of the electrons (1.9 10^7^ m.s^-1^ for 1 keV) and w the half width of the electron packet. With a target of 0.05 i.e. 10 mm deviation and w at 50 μm taking into account that plates are located at the focal point of the Einzel lens, we can achieve a shearing limited temporal resolution of 0.5 ps. In turn, these values allow to use nearer and smaller parallel plates (d=0.5 mm and L=0.05 mm), that are much easier to charge and increase the bandwidth of the streaking system. Under these conditions the deflection ramp that can be achieved is typically of 1 V.ps^-1^. However, in practice, the beam size on the MCP is in the mm range, which practically limits the time resolution to about 5 ps (equivalent to two pixel uncertainty in the figures of Table. S5).

| Relative UV intensity | Charge | Direct Image | Deconvolution | τ (ps) |
| --- | --- | --- | --- | --- |
| 1 | 80±4 fA  (500±30 e-) | 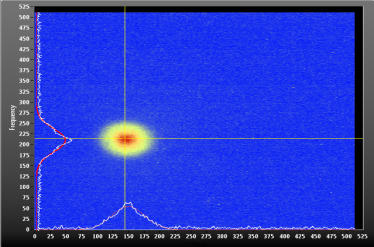 | 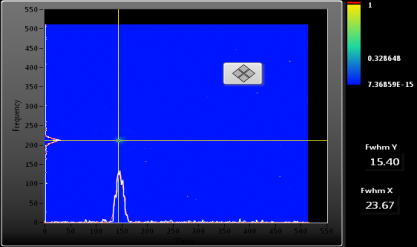 | 18±5 |
| 10 | 0.9±0.05pA  (5600±350 e-) | 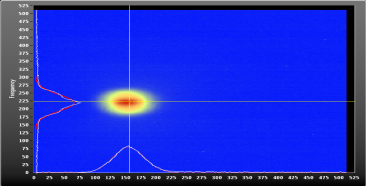 | 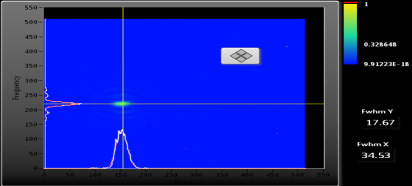 | 30±5 |
| 100 | 6.5±0.33pA  (40000 ±2000 e-) | 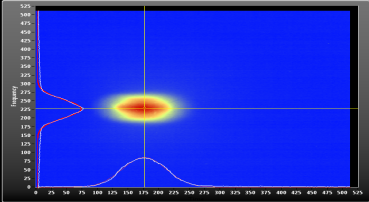 | 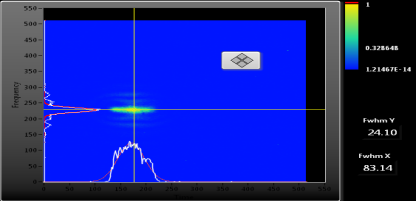 | 70±5 |

*Table S5: Typical results from the streaking at the ELYSE platform.*

The packet lifetime measured in table S5 can be expressed as.

$$\tau= \sqrt{{\tau_{UVpulse}}^{2}+{\tau_{coulombic-repulsion}}^{2}+{\tau_{energy-dispersion}}^{2}+ {\tau_{high-tension}}^{2}}$$

From the litterature ^2^, ${\tau_{coulombic-repulsion}}$ is proportional to the square root of the number of electrons N in the bunch, but also depends on the distance between the electrons which is first governed by the distance between photoionization sites on the photocathode. It is dominant for bunches with high N (as verified from the shape of the curve of figure 2) but it is not expected to impact the bunch duration for the very small values of N used in the cathodoluminescence experiments.^3^ Indeed, SIMION simulations *in the absence of initial energy spread* such as reported in figure 2 give a duration of 2 ps for 100 electron bunches in our geometry.

$\tau_{UVpulse}$is about 100 fs

$\tau_{high-tension}$is connected to the uncertainty of the calibrated power supply, here 10^-5^ of the setted HV value. For a time of flight of about 20 ns to reach the detector at 1 keV, it represents 0.2 ps fluctuations.

$\tau_{energy-dispersion}$ accounts for the effect of the initial spread in electron energy onto the time of flight of the electron. This impact can be evaluated by SIMION simulations *in the absence of coulombic repulsion* with our experimental geometry*,* For an initial energy spread of 0.1 eV ^4^and an accelerating voltage of 1kV, it represents 17 ps of difference of time of flight at the measurement point (see the blue line reported on figure 2). This value decreases to 4 ps for a 4 kV accelerating voltage

So the observed electron bunch duration is dominated by the energy spread effect for the very small values of N.

The uncertainty on the charge was evaluated from the precision of the current measurement (5% according to the electrometer manual) and the accuracy of the frequency measurement (GHz oscilloscope, 1% precision).

**A.3. Dose calculation**

The dose rate on the sample was calculated using the beam current, the acceleration voltage, the beam size measured above and CASINO software simulation of the penetration depth ^5^.

Fig S6: Energy deposited as a function of depth by 4.1 keV electrons, as determined by the “cathodoluminescence” intensity data from CASINO simulations ^5,6^. The DNA density was taken from ^7^. The integration of the different part of this curve allowed to calculate the proportion of electron energy effectively reaching the DNA film.

The amount of irradiated matter was evaluated to 5*10^-5^ mg with the electrons loosing 95 % to 99 % of their energy on the first 40 nm of gold. For one second irradiation time at 1.2 nA, the energy received is in the range of 250 to 50 nJ. The dose rate expected on the sample is therefore in the kGy.s^-1^ range.

**B. Time-resolved luminescence data for BC-422Q**

B

A

Fig S7: A) TR-PL of the BC-422Q scintillator and B) TR-CL of the same scintillator

B

A

*Fig S8 :* *BC422Q luminescence data (in black) and their adjustment (in red) by an addition of three exponential decays A) TR-PLτ1=100 ps, τ2=400 ps and τ3=1900 ps., B) TR-CL τ1=80 ps, τ2=380 ps and τ3=1000 ps*

Fig S9: BC-422Q cathodo (CL) and photo (PL) luminescence intensity data.

**C. Evaluation of excitation yields and associated uncertainties**

The scintillation yield of DNA Φ_sci-dna_ can be expressed as :

Φ_sci-dna_= Φ_sci-BC422Q_ ÷ CE_dna/BC422Q_ × NP_dna/BC422Q_ = Φ_sci-anthracene_ × RE_BC422G/anthracene_  ÷ CE_dna/BC422Q_ × NP_dna/BC422Q_

Where NP_dna/BC422Q_ is the relative number of photons emitted by DNA compared to BC422Q in the same irradiation conditions; CE_dna/BC422Q_ is the relative photon collection efficiency of the DNA sample compared to the BC422 scintillator; RE_BC422G/anthracene_  is the relative scintillation efficiency of the BC422Q compared to the reference anthracene and Φ_sci-anthracene_ the anthracene scintillation efficiency

NP_dna/BC422Q_ was evaluated to (16±1)% by integration of the time resolved scintillation data without filter.

RE_BC422G/anthracene_  is given by the manufacturer , but without any incertainty of the value. However, the precision on the measurement given in the specification sheet are at the % level. Therefore, we took the scintillation efficiency of the BC-422Q to (4±1) % of that of anthracene. This is obviously the main source of incertainty.

The scintillation efficiency of anthacene Φ_sci-anthracene_ is measured in the literature at 1 photon emitted for 55 eV (±10%) ^8^

The collection efficiency CE_dna/BC422Q_ was evaluated using Fresnel equations with the following optical data : optical index of BC422Q, 1.580 ^9^ optical index of the sapphire 1.768, ^10^ optical index of the DNA 1.585 ^11^ optical index of CTAB 1.435 ^12^ and a numerical aperture of 0.4. The CTAB DNA layer index was calculated following ^13^ as 1.5

For the DNA/sapphire assembly, the optical losses dues to interfaces are evaluated to 8%, and the collection angle decrease from de 23.5 to 16.5 degrees

For the BC422Q, the optical losses dues to interfaces are evaluated to 5 %, and the collection angle to 15.5 degrees. The relative variation in collection angle leads to a 6% increase in collection efficiency in favor of DNA, as the scintillation is expected to be isotropic. Therefore, we took CE_dna/BC422Q_ as 1.03 , with an error associated of 5% at the most.

These values lead to a scintillation efficiency of 0.012 photons for 100 eV, with an associated error of 50%

**References**

1 Jaanimagi, P. A. *Breaking the 100-fs barrier with a streak camera*. Vol. 5194 OP (SPIE, 2004).

2 Tao, Z., Zhang, H., Duxbury, P. M., Berz, M. & Ruan, C.-Y. Space charge effects in ultrafast electron diffraction and imaging. *Journal of Applied Physics* **111**, 044316, doi:10.1063/1.3685747 (2012).

3 Wytrykus, D. *et al.* Ultrashort pulse electron gun with a MHz repetition rate. *Appl. Phys. B-Lasers Opt.* **96**, 309-314, doi:10.1007/s00340-008-3355-1 (2009).

4 Janzen, A. *et al.* A pulsed electron gun for ultrafast electron diffraction at surfaces. *Review of Scientific Instruments* **78**, 013906, doi:10.1063/1.2431088 (2007).

5 Drouin, D. CASINO a Powerful Simulation Tool for Cathodoluminescence Applications. *Microscopy and Microanalysis* **12**, 1512-1513, doi:Doi: 10.1017/s1431927606069686 (2006).

6 Demers, H., Poirier-Demers, N., Phillips, M. R., de Jonge, N. & Drouin, D. Three-Dimensional Electron Energy Deposition Modeling of Cathodoluminescence Emission near Threading Dislocations in GaN and Electron-Beam Lithography Exposure Parameters for a PMMA Resist. *Microscopy and Microanalysis* **18**, 1220-1228, doi:10.1017/s1431927612013414 (2012).

7 Śmiałek, M. A., Jones, N. C., Hoffmann, S. V. & Mason, N. J. Measuring the density of DNA films using ultraviolet-visible interferometry. *Physical Review E* **87**, 060701, doi:10.1103/PhysRevE.87.060701 (2013).

8 Wright, G. T. Absolute Scintillation Efficiency of Anthracene Crystals. *Proceedings of the Physical Society. Section B* **68**, 929 (1955).

9 *Plastic scintillators, BC422Q* [*https://www.crystals.saint-gobain.com/products/bc-418-bc-420-bc-422-bc-422q*](https://www.crystals.saint-gobain.com/products/bc-418-bc-420-bc-422-bc-422q).

10 Malitson, I. H. Refraction and Dispersion of Synthetic Sapphire. *J. Opt. Soc. Am.* **52**, 1377-1379, doi:10.1364/JOSA.52.001377 (1962).

11 Inagaki, T., Hamm, R. N., Arakawa, E. T. & Painter, L. R. Optical and dielectric properties of DNA in the extreme ultraviolet. *The Journal of Chemical Physics* **61**, 4246-4250, doi:10.1063/1.1681724 (1974).

12 Kekicheff, P. & Spalla, O. Refractive Index of Thin Aqueous Films Confined between Two Hydrophobic Surfaces. *Langmuir* **10**, 1584-1591, doi:10.1021/la00017a043 (1994).

13 Heller, W. Remarks on Refractive Index Mixture Rules. *The Journal of Physical Chemistry* **69**, 1123-1129, doi:10.1021/j100888a006 (1965).
